# Optimising the public health benefits of sex work regulation in Senegal: Results from qualitative interviews with policy stakeholders

**DOI:** 10.1371/journal.pone.0306803

**Published:** 2024-08-15

**Authors:** Aurélia Lépine, Fanny Procureur, Sandie Szawlowski, Carole Treibich, El Hadj Mbaye, Khady Gueye, Cheikh Tidiane Ndour

**Affiliations:** 1 Institute for Global Health, University College London, London, United Kingdom; 2 CNRS, INRAE, Grenoble INP, GAEL, Univ. Grenoble Alpes, Grenoble, France; 3 AIDS Division, Ministry of Health and Social Action, Dakar, Senegal; University of KwaZulu-Natal College of Health Sciences, SOUTH AFRICA

## Abstract

**Context:**

There is compelling evidence that eliminating sexually transmitted infections (STIs) among female sex workers (FSWs) is a cost-effective approach to reducing the spread of HIV/AIDS. Although many countries recognise sex work as a public health issue, few have implemented public health policies specifically aimed at controlling the transmission of HIV/AIDS among FSWs. In particular, Senegal stands out as the only African country to regulate sex work through a specific public health policy that requires FSWs to register with a health centre. Despite the potential health and legal benefits associated with registration, a staggering 80% of FSWs in Senegal remain unregistered. This low registration rate hinders the realisation of the policy’s full potential for public health benefits. The reluctance of FSWs to register is due to inherent flaws in the policy design, where the disadvantages of registration outweigh the benefits for FSWs.

**Objective:**

To identify which modifications to the current registration policy have the potential to increase uptake of registration by FSWs and to assess their feasibility in the context of Senegal.

**Method:**

We conducted a qualitative policy research study using semi-structured in-depth interviews with 22 national stakeholders in this policy, including representatives from the police, government and non-governmental organisations (NGOs) in Dakar, Senegal, as well as FSWs’ leaders. The interview data were thematically coded using the interview topic guide and other recurring themes and analysed using thematic analysis on Nvivo 12.

**Results:**

A total of 20 relevant themes were selected, focusing primarily on assessing the feasibility of potential interventions and identifying potential barriers and associated risks. We found that, without changing current legislation, improving relationships between FSWs and police officers, providing accurate and accessible information about the rules and benefits of the policy, and offering psychosocial support have the potential to improve both the registration rate of FSWs and their wellbeing. Policy features designed to increase registration by improving FSWs’ confidentiality, and thus their confidence in the services offered, were also discussed.

**Conclusions:**

The study highlighted that several national public health policies could be changed to increase the registration rate of FSWs and improve their wellbeing without overturning constitutional law.

## 1. Introduction

Globally, robust evidence supports the notion that eradicating sexually transmitted infections (STIs) among female sex workers (FSWs) constitutes a cost-effective strategy for mitigating the spread of HIV/AIDS [1]. In West Africa, the HIV epidemic is concentrated within the FSW community, with 75% of male HIV infections directly attributable to sexual interactions with FSWs [2]. Senegal, specifically, reports a significantly heightened risk of HIV infection for FSWs—16.5 times greater compared to the general population [3].

While many countries recognise sex work as a public health issue, few have adopted the policy of registering FSWs and monitoring STIs as a proactive measure to control the wider spread of HIV/AIDS. Notable among these is Senegal, the only African country that currently regulates sex work through a comprehensive public health policy, a legal framework established in 1969. (cf. Code pénal articles 319/ 325).

The current registration policy originates from the French colonial era and is rooted in the medical and sanitary policies established at the federal level until Senegal gained independence in 1960 [4]. In the second half of the 19th century, the serious impact of syphilis, particularly in colonial cities and among military personnel, became a major concern for colonial authorities. Soldiers were hospitalised and the overall effectiveness of the military was compromised. At the time, medical authorities believed that the main source of syphilis transmission was associated with sex work. The prevailing belief was that syphilis was primarily spread through sexual intercourse, and as a result FSWs were often singled out as the perceived source of syphilis transmission [4]. This understanding led to the implementation of public health measures in 1849 to minimize the spread of STIs among this population [5]. A decree was issued requiring FSWs to undergo compulsory health checks. The decree required them to register with a hospital and carry a health booklet.

In 1946, sex work was outlawed by colonial authorities, and all records of FSWs were reportedly destroyed. However, the Senegalese Minister of Home affairs at the time held the view that regulating sex work might be a more effective strategy than outright prohibition [6]. Consequently, following independence, the Senegalese Minister of Home affairs endeavoured to legalise sex work and reinstate the registration policy. More than two decades after its initial abolishment, in 1969, the registration policy was reintroduced, requiring Senegalese FSWs aged 21 and above to register with a health centre undergo routine monthly health visits. The regulatory framework established in 1969 has undergone minimal changes since its implementation, with adjustments primarily related to the appearance of the registration booklet. This policy mandates compulsory testing, treatment in the event of a positive diagnosis for a STI, and the provision of free condoms [7]. Registration entails the issuance of an official booklet referred to as the "carnet sanitaire", which functions as a record of a FSWs visits to the healthcare centre. In cases where FSWs test positive for any STI, excluding HIV, the booklet is retained at the health centre throughout the entire treatment course. FSWs diagnosed with HIV are directed to additional care, with adherence to antiretroviral treatment monitored during routine visits. Possession of an updated registration booklet is a prerequisite for FSWs to engage in legal sex work. Failure to present this booklet—whether due to non-registration, non-compliance with routine visits, or ongoing treatment for an STI—may lead to imprisonment, with a potential sentence ranging from 2 to 6 months (cf. Code pénal articles 319/ 325).

Despite the potential advantages associated with the registration policy, estimates indicate that approximately 80% of FSWs in Senegal, and 57% specifically in the capital city of Dakar, remain unregistered [3]. The primary obstacle to registration is recognized as the stigma associated with sex work, wherein the apprehension of social ostracization emerges as a predominant barrier [8].

In 2015, a cohort study encompassing 600 FSWs in Senegal—300 registered and 300 non-registered FSWs—enabled the first assessment of the impact of the registration policy, aiming to elucidate the factors contributing to the low registration rate [7]. The study revealed that the registration of FSWs led to a 38% reduction in the prevalence of STIs, underscoring the efficacy of the registration policy in encouraging high-risk populations to utilize services for STI prevention and treatment. However, the study brought to light two significant challenges associated with the current policy. Firstly, despite its legal status, sex work is socially condemned in Senegal, instilling a pervasive fear among many FSWs that the revelation of their activities could lead to social ostracism. This stigma plays a key role in fostering hesitancy among FSWs to partake in activities that might elevate the risk of their engagement in sex work being exposed to others. A substantial 56% of FSWs cited the registration proof as the primary reason for non-registration. This reluctance is rooted in the obligation for FSWs to carry and conceal their registration card to legally practice sex work, making them easily identifiable and thereby increasing the risk of discovery by family or friends. Additionally, the study noted that mandatory routine health clinic visits for registered FSWs are concentrated on a specific day (Thursdays), heightening the possibility of other patients discerning the purpose of their appointment. Moreover, even if registered FSWs cease engaging in sex work, their personal information is often retained indefinitely in police and health centre records.

Secondly, registration was found to detrimentally impact FSWs’ mental health and well-being, which can lead to riskier health behaviours. This was also highlighted in other studies showing that criminalisation tends to isolate and stigmatise FSWs and their clients [9–12] and this can actually result in more risky behaviour and higher rates of STI transmission [10, 11, 13, 14]. Key changes to the current policy design are therefore needed to increase its effectiveness and further reduce the spread of STIs among FSWs and in the general population.

In 2020, as part of the third wave of the cohort study, a discrete choice experiment (DCE) was completed by 241 registered and 273 non-registered FSWs to identify the policy elements that are either appealing or unappealing to them as participants were required to make choices between a series of hypothetical policy changes [15]. In addition, the DCE methodology made it possible to estimate the potential uptake of registration for a policy that combines key components. The results showed that several policy adjustments hold promise for increasing the registration rate among FSWs and improving their overall well-being, all within the confines of existing legislation. For example, switching from the traditional proof of registration to a QR code format and including psychosocial support in the registration package were identified as measures that could increase the benefits of registration. In addition, integrating mandatory sexual health appointments for registered FSWs with maternal health appointments and strengthening confidentiality training initiatives at health centres emerged as strategies with the potential to encourage registration among FSWs through stigma reduction.

The objective of this study, operating within the current socio-political environment, is to assess the feasibility of implementing promising modifications to the registration policy identified by the DCE. These modifications are aimed at increasing the registration rate among FSWs. The remainder of this paper is structured as follows: Section 2 details the methodology, Section 3 presents the results, and Section 4 undertakes the discussion. The paper concludes in Section 5.

## 2. Methodology

The qualitative research was conducted in December 2020. The study also engaged policy stakeholders in the Dakar region of Senegal with extensive contextual knowledge and experience in supporting the conduct of research projects.

A series of qualitative interviews with key informants were conducted: officials from the Ministries of Health and Home Affairs, doctors and social workers responsible for routine visits to FSWs, police officers, NGO staff working with FSWs and FSWs’ group leaders.

We included four categories of participants in our sample (see S1 Appendix): district doctors (n = 4), nurses/midwives (n = 4), government representatives (n = 6), NGO leaders (n = 3), senior members of the police (n = 2), and FSWs’ group leaders (n = 3, 1 registered and 2 non-registered FSWs). The group leaders are FSWs with extensive knowledge of the profession. They actively support and represent both registered and unregistered FSWs. Consequently, their perspectives are shaped by a synthesis of their own personal encounters within the profession and the collective experiences of FSW under their advocacy and support. In total, 22 participants were interviewed.

Participants were purposefully selected for the study. Recruitment of both medical and non-medical staff was guided by their engagement in providing services to FSWs. Most participants were chosen by the research team with the support of the HIV division at the Ministry of Health (DLSI) by leveraging existing relationships cultivated during the PI’s prior involvement in a FSWs’ cohort study or through government partnerships established by the project. The police represented the only group with whom there was no prior relationship, but engagement was facilitated by the DLSI. The nature of the relationship between the researchers and participants remained strictly formal and work-related, with no personal information exchanged. Initial contact with all participants was by telephone and all those contacted volunteered to take part in the study, with no refusals or drop-outs.

Interviews took place at the participants’ respective workplaces. Interview sites included hospitals, health centres, HIV care facilities, police stations, government buildings, and NGOs. For FSWs’ group leaders, interviews were conducted in private rooms to maintain confidentiality. No individuals besides the researcher(s) and the participant were present during the interviews.

Prior to each interview, the researchers presented a number of suggestions for interventions and policy changes that have been shown to improve registration rates in the DCE to interviewees [15]. These included altering the appearance of the registration card to enhance discretion and reduce stigma, for instance, by implementing a QR code. Additionally, suggestions involved the introduction of psychosocial support, changing the format of the compulsory medical visits, and enhancing communication and information dissemination among FSWs, police, and health services. Hence, the interview topics outlined in the defined interview topic guide encompassed perspectives on registration laws, experiences in interacting with FSWs, and the practicality of potential policy changes to further understand the feasibility of the findings from the DCE research.

Prior to the interview, all participants received information regarding the research objectives and anticipated outcomes. Each participant was provided with an information sheet detailing the study’s commitment to maintaining confidentiality and their prerogative to discontinue the interview or withdraw from the study at any juncture. Additionally, participants were required to read and sign a consent form prior to engaging in the research. Participation in the survey was voluntary and unpaid. Interview durations ranged from 20 to 70 minutes. Except for one interview, all sessions were recorded using audio recording equipment. One of the participants expressed reluctance to be recorded, and in agreement with them, a researcher took detailed notes throughout the interview process. To preserve anonymity, all names mentioned in the interviews were replaced with ’X’. No repeat interviews were conducted.

During and after interviews, both researchers documented fieldnotes to aid in reflection and data triangulation. Subsequently, recorded interviews were transcribed, cleaned, and stripped of any potentially identifiable information. Transcripts were not returned to participants for review or correction. The qualitative data underwent discourse analysis, facilitated by thematic analysis using Nvivo 12 software. Initial thematic categories were derived from a literature review and insights from local collaborators regarding the FSW registration policy. Any emerging or recurring themes identified during analysis were incorporated into the thematic framework. Furthermore, connections between main themes and sub-themes were explored to offer new perspectives on the data. All coding and analysis was conducted in French. Select quotes were translated from French to English to elucidate the themes and findings of the study. Each quotation was accompanied by the participant’s role, such as Member of Ministry of Home affairs or Hospital social worker. Notably, the diverse experiences of group leaders among FSWs with varying registration statuses were highlighted, with their registration status and participant number noted.

Participants did not provide feedback on the findings; however, a policy brief was crafted incorporating the research findings and disseminated to governmental stakeholders.

### 2.1 Ethical statement

This research was granted ethical clearance by the University College London Research Ethics Committee on 12 May 2020 (Project ID: 17341/001) and by the national ethics committee in Senegal. An information sheet was distributed and written consent was obtained for each participant.

## 3. Results

The key themes discussed were discerned from the previously conducted DCE, the interview topic guide and recurring patterns that emerged throughout the interviews. These overarching themes encompassed the reasons for the low registration rate, the viability of potential interventions (e.g., changing registration proof, modifying the format of medical visits, and offering psychological support), as well as any associated risks or structural impediments. Sub-themes were subsequently formulated to provide substantiation and further detail for each thematic category.

### 3.1. An obsolete but ingrained law

Most respondents reported that the FSW registration law was outdated, tracing it back to colonial times. Respondents felt that this law was no longer appropriate to the current situation. However archaic this law is, respondents did recognise that even if many policy actors still do not understand or fully support this law, it was still well ingrained and deeply rooted in society. Going to war against it could be politically risky and could cost people their reputation. Indeed, obtaining a majority of votes to modify the law by the Senegalese parliament simply feels impossible in the current context.

*«People in parliament… it will be all over the media, that such and such person is promoting prostitution in Senegal. »* Member of Ministry of Home affairs

*« We held a national advocacy workshop*: *there were members of the parliament*, *police officers but the problem is*… *everyone knows texts are obsolete but no one will be able to carry this fight*. *Nobody*. *Talk about prostitution in Senegal*? *It’s sensitive*! *There are religious*, *socio-cultural considerations*. » Hospital social worker

*« You would need 80 people’s votes for the new law to pass*, *because we have 160 members of the parliament*. *So*, *it is not enough that there is just someone who can bring the change in the law at the parliament*, *it will be necessary that this person manages to convince many members of the parliament If it’s a state law*, *it would be passed because the state has a majority in the parliament*, *because these would have to be recommendations from the palace* (government) *and the Minister of Health can’t do anything on its own*. » Member of Ministry of Home affairs I

Many of our respondents think that the main reasons why most FSWs do not register are not only the inadaptability of the law and need for anonymity, but the new emerging types of sex work, which are developing outside this regulated system.

*«Sex work has developed a lot outside the system, FSWs who do not register are even more numerous. Today young girls rent apartments, contact clients on social media, all of this can be done without having to register or without the police or the local doctor knowing it; registration is no longer a constraint for a woman who wants to sell sex.* » NGO director_1

*«They prefer to be illegal at the risk of being caught by the police; also*, *in the end*, *those registered run the same risks as the illegal ones*. *So*, *this is the blocking factor for the registration of FSWs*. » Doctor_1

In addition, the current law does not allow for the solicitation of clients. Therefore, even registered FSWs are illegal if they solicit clients.

“[This law] *has never changed or evolved compared to the current context: because by digging deeper, we see that even if* [sex work] *is allowed, the place where they work is already a problem. If you are found in the street and you are soliciting clients, it is illegal. If it is in hotels and bars, they say you are ‘soliciting clients’; so where will they work? Either we remove the law and ban sex work, or we improve the working conditions of these FSWs.*” Doctor_1

*“There are a lot of things that need to be amended in this law*, *which in fact simply tolerates sex work*. *Indeed*, *we give you a card but no place to work*, *that does not make any sense*. *We give you one hand but we take away the other hand”*. Member of police

### 3.2. Misinformation

We found that many FSWs are not registered because of misinformation stemming from rumours or preconceptions of the registration policy. Respondents mentioned that a lot of FSWs believe they will no longer be able to travel or have certain occupations if they have registered as an FSW. In addition, procedures regarding their files and storage of their personal data are not explained to FSWs when they first register, which naturally fuels their fear and anxiety.

*«No when we register, we are not told anything. They just talked about illnesses, and the police.* » FSW_1 (registered FSW)

*«Those who do not go to register are afraid*. *A card*? *I wanted one and got it but now I regret it because you’ll get to a point where you can’t travel so you’re stuck*. *And the day your children grow up and want to go on a trip*, *they will have problems doing so*. » FSW_1 (registered FSW)

*«The day your child wants to study and we see his mother’s profession*, *this can cast a bad shadow on him*. *When you have a card*, *you are therefore stuck*, *with everything that comes with it*. » FSW_2 (non-registered FSW)

*“In any case my biggest question is what do they do with these papers afterwards*? *I’ve asked this question many times but I can’t get a clear answer*. » *FSW*_2 (non-registered FSW)

Finally, different profiles of our respondents said that it is still unclear whether the FSW’s file remains archived at police level once they leave sex work or whether it is destroyed. In cases where participants believed it was archived, they did not know who could access these files. All of these issues place a huge burden on FSWs and act as a strong deterrent to registration.

*«They tell you to come back when you want to leave sex work, with your documents to be removed from the file but when that time comes, apparently they actually don’t give those documents back to you.* » FSW_1 (registered FSW)

*«We are only told that files will be returned to the police [if they leave sex work]*, *they do not explain the process to us*. *At the police level*, *I really don’t know if they are destroyed; and I don’t know if files are well kept*. » Sex work hospital mediator_1

From the police’s point of view, radiation (complete riddance of physical file) from police files seems to be relatively easy, even if procedures seem extremely long and burdensome.

*«Her quitting sex work will be investigated. First at the key population hospital level to find out if this FSW has effectively left sex work. Then we do a ‘enquête de moralité’. If all this is done, we remove her from the files: we come to the health facility, we make a report on the basis of what she said, we make her read and sign.* » Police representative

An ‘enquête de moralité’ is a legal procedure conducted by the police, involving background checks on FSWs who have indicated their intention to leave sex work, aimed at confirming the cessation of such activity.

### 3.3. Change in registration proof

Most respondents felt that the replacement of the physical card by a QR code could be a good idea which would solve many issues linked to confidentiality, mental health, and medical follow-up. Many FSWs mentioned that discretion and confidentiality would be improved with the implementation of QR codes. Moreover, removing their name and the “sanitary card” reassured them vis-a-vis their family and close relatives. This finding is to be expected, given that the discovery of their occupation by their relatives was a major barrier to registration.

*«It is practical, much more discreet and it’s impossible to know what it is.* » FSW_2 (non-registered FSW)

*«I could tell my children that this QR code is the thing I use to make payments* [since many banks are using this technology in Senegal], *and it would no longer be a problem to carry it*. » FSW_1 (registered FSW)

Other respondents expressed their support to replace the physical card with a QR code given that its introduction would be relevant in a society like Senegal, currently transitioning into a digital era. We also found that the QR code was viewed as a safer way to store information, not only because of its confidentiality but for its intangible character, meaning invisible but also less difficult to misplace.

*«It is the future (…) it is absolutely in line with the current evolution of the world, this digitalization, this use of intangible resources; I find that to be very good.* » NGO director_1

*«Right now*, *there are doubles*, *even triple registrations per FSW*. *You find the same person*, *registered in five health care centres*. *You understand*? *Sometimes you think of her as lost to follow-up when she simply left the area*. *So*, *we need to get digitalised health files*. » NGO director_2

*«This is the reason why we think they are “lost"*, *but they just change places; and start over again with a new doctor*, *going over it all again is so laborious*. *It would be easier to have a process that allows you to have everything stored in one place*. » Doctor_1

Lastly, another advantage would be that it would prevent corruption. It is observed that policemen sell fake up-to-date health booklets to FSWs. QR codes would be much harder to falsify that the booklets for policemen and this in turn would limit potential bribes.

*«Policemen told us that the national health card was not their problem but that women would have to buy their own registration card at CFAF 6,000 (~USD 10). They said it was a mean to count sex workers.* » Member of Ministry of Home affairs

In terms of feasibility, most respondents expressed their concerns regarding resources needed for the implementation of these changes (e.g., internet access in health centres and QR code scanners). Except from this logistics challenges, many stakeholders felt that this change was implementable and would be well received by the police and health staff.

*«I think it’s possible, the police force of Senegal is very well equipped. They have a lot of equipment they are used to; the other thing, recently they made new biometric ID cards and I am sure that in some time they will use devices to scan, so we are going digital, that’s for sure*. » NGO director_1

*«If our hierarchy accepts this strategy*, *because they need to*, *they just have to confirm with the police on the ground*, *train them and then put them to work*. » Police representative

*«Yes*, *we think that if the means allow it*, *it will be less expensive to provide each police station with a scanner and training the officers on how to use it*. *The police only obey the directives of the hierarchy*. » Senior police officer

### 3.4. Confidentiality at health centre level

The lack of confidentiality is well-known to be main barrier to registration for FSWs. This is because FSWs fear that their file held at the health centre could end up in the wrong hands.

*“Sometimes they ask you this question: "is it safe to leave the file here? Later will my child not be in contact with this file, or his friends…? These files, we only work on them in here; even if their son or sister comes and asks us for information, they won’t get it because it’s our ethic. It does not concern them; they won’t get any information from me. That’s it.” Midwife_2*

In reality, the fear of confidentiality breaches stems from the fact that many health centres organise the mandatory sexual health visits for FSWs on Thursdays. In recent years, increased efforts to integrate FSWs’ routine visits with maternal and women’s health visits have made it more difficult for people to determine that the woman they see at the health centre is an FSW. This leaves the physical registration booklet as the only identifier that she is engaged in sex work. However, this booklet is sometimes difficult to hide during health visits. In general, the medical staff we spoke to mentioned that they take extra care to protect identities or make extra efforts to see FSWs quickly, reducing the time they spend waiting in health centres, in order to avoid social stigma.

*«Here, they are included in the women’s health consultations. We used to see them in the referral key population hospital* [called Institut d’Hygiène Sociale], *but we changed, precisely to avoid social stigma.* » Doctor_1

*«What matters to us is that you can come and make your visits without anyone knowing anything*, *in all discretion*, *like any other patient*. *Even if you turn out to be positive on a visit*, *they know where to point you without anyone knowing*. » *FSW*_1 (registered FSW)

*«Confidentiality is built into all hospital wards*. *Because at our level*, *if we do the consultations this way*, *by mixing them with other women*, *it is precisely to avoid stigma*. » Nurse_1

This fear of lack of confidentiality at the hospital is heighted with the fear that health centres communicate with the police and exchange files and information of FSWs. According to our respondents, this finding was unfounded. Rather, we found that communication between the two institutions (police and hospital) was poor and sometimes even non-existent. However, none of our respondents had a clear idea if there was a process through which the police could demand certain health files to the hospital.

*«Between the hospital and the vice squad of the police? Oh no, really at our level, there is no collaboration.* » Midwife_2

*«No*, *I never had any contact with them [police officers]*, *they never asked me for information; I don’t even know them*. *I guess they would need to follow a procedure to access a file*. » Midwife_3

All respondents did mention that files stored at the hospital were systematically archived and not destroyed, following the same process as all other medical files. A reason for this mentioned by health staff was that most FSWs leave sex work but the majority return to sex work at some point. Hence, destroying their file would mean losing their medical history.

*«You cannot destroy the file instantly because over 90% of FSWs leave and come back afterwards, and when they come back you have to reconstruct it and you will have lost all the medical data on their visits and their health during all the time that they were there. You lose it all.* » Social worker_1

*«In fact*, *even I said I quit and then came back; when I said that I had come back*, *my file was immediately pulled out to say that there you are*, *and I had the same number as before*! » FSW_1 (registered FSW)

Taking all of this into account, FSWs still expressed their doubts regarding confidentiality at the hospital. This doubt was mainly motivated by the potential for hospital visitors to identify them and expressed a general preference for more discreet easy access mobile clinics or private doctors.

*«I’ve seen a lot of FSWs who left conventional health sites to see private gynecologists. Examinations and check-ups, you get results, you get treatment. If you have a permanent partner, you bring him in and same treatment: and you are in good health…So she is better followed than the SWs at the hospital! So perhaps the Government should contract with the private sector; and there, we come in confidence.”* FSW_3 (non-registered FSW)

### 3.5. Healthcare costs

The current policy demands that health visits are paid for. For this reason, we investigated the rationale and feasibility of free health care visits to understand whether cost was a contributing factor to the low rate of registration.

*«In my opinion they are concerned about their health and no matter the price they will come, the problem is not the money but the registration card; or care that is not adequate, but it is not a question of money.* » NGO director_1

*«Money isn’t the problem*, *FSWs make a living*. *We’d come even if it was CFAF 20*,*000 [10 times greater than current price]*. *When there is security*, *confidentiality*, *it is better for us*! *It is more important to be safe and to be sure that our information is well kept*, *that there will be no stigma or discrimination and thus we will be more comfortable doing our consultations*. » FSW_3 (non-registered FSW)

### 3.6. Psychological support

Offering psychological support, as part of the registration policy package, to FSWs was investigated as the literature notes that FSWs often feel depressed and experience many traumatic events [16–18]. Psychological support is generally hard to access or non-existent for FSWs. The lack of psychologists and resources make it difficult to create a dedicated mental health space for FSWs. In this context, midwives and social workers or even sex work group leaders are usually the ones who take up the role of a psychologist.

*«I asked her why she drank so much alcohol, she replied “because when I am conscious, I cannot do it, I have to be unconscious because my head is telling me you must not do that, you are a Muslim, if your children find out that this is what you are doing to feed them, they will hate you…” I seriously think they need it. It’s difficult, and there is also all the harassment, this stigma that is out there!* » NGO director_1

*«So*, *we do it ourselves*. *When there is a sex worker calling me who is infected*, *even at night there are some who wake me up because they are not sleeping*, *and they are upset*. *You know they suffer a lot of violence*, *sometimes it’s the client*, *other times the boyfriend*. » Social worker

*«Usually*, *it’s with social workers and physicians who care for them*, *because we don’t have psychologists*. *It’s only in Dakar that you could get your hands on a psychologist but they are rare*. » *Doctor_1*

*«Of course*, *it is feasible*. *For me*, *hospital is where it [psychological support] belongs*, *it must be part of the services offered (part of the registration policy)*, *to see the state of their mental health*, *all that*. *And see how we can refer them to someone*. » *NGO director_1*

### 3.7. Interaction with police

The relationship with the police was reported to be tense and difficult in many ways. Despite a lot of work to raise awareness and dialogue with the police, most of our respondents confirmed that there is still a lot of abuse by police officers against FSWs. This took the form of psychological and physical violence, extortion, bribery, blackmail, arbitrary arrest, spending the night in jail without charge and breach of confidentiality.

*«Once there was a raid and she was asked to pay the FCFA 5000 (~USD 8) as usual (…) Yes, it is like this every night… she said no and the policeman beat her up.* » Social worker

*«Sex workers hide their profession; because if you’re known to the police*, *even if you are not working at that point*, *just being out gives them the right to arrest you when and how they want*. » FSW_1 (registered FSW)

*«Girls can go and file a complaint against police abuse*, *but sometimes the police will say it’s not worth it because tomorrow these same people will still be on the ground and there is the risk of reprisals*… ». FSW_3 (non-registered FSW)

These relationships tend to be even more problematic and complicated given that many FSWs know and work directly with policemen, either by having them as clients or by buying their protection in exchange of sex acts.

*«Some FSWs have policemen’s phone numbers. Sometimes yes, they are clients, boyfriends, collaborators too.* » Social worker

*«Yes*, *FSWs have sexual encounters with policemen*. *Some are clients*, *they pay*. » Sex work hospital mediator

*«I see policemen who go out with sex workers*, *I saw them*. *They even marry them; I don’t know if it is their relationship that leads to violence*, *God knows what happens in raids at night*… *I myself was a victim of that*: *police officers treated me badly*. » Police representative (female)

Recently, FSWs who are victims of abuse have benefitted from free legal counselling services in Senegal, called “les boutiques de droit”, which our respondents considered to be a considerable source of help and support.

*«We referred one woman to the “boutique de droit” (because she was raped), she then took all the necessary steps. She filed a complaint, met with the prosecutor. And there is the possibility of having a closed hearing, which gives them much more courage.* » Midwife_4

## 4. Discussion

Senegal is the only African nation where sex work is legalised and regulated. This study endeavours to explore the prevailing challenges faced by FSWs in Senegal while identifying opportunities to improve the current registration policy.

The study unveiled that the removal or any substantial change in the sex work law would be politically costly in terms of reputational risk. Consequently, even if proposed amendments to the law are introduced in parliament, the likelihood of garnering a majority of votes remains low. This phenomenon is not unique to the domain of sex work legislation in Senegal; rather, it extends to other sensitive policies on a global scale. Evidence from various jurisdictions and contentious policy areas supports the notion that politicians often face reputational risks when advocating for the removal or substantial alteration of established policies [19].

The interviews revealed that many of the concerns discussed arose from the stigma associated with sex work. The highlighted issues, encompassing misinformation or a dearth of information, the importance of confidentiality of FSW, dynamics between FSWs and the police, as well as challenges pertaining to mental health and well-being, all find their roots in FSWs wanting to keep their profession hidden from family and friends for fear of social ostracization.

First and foremost, the preservation of a FSWs confidentiality emerged as a crucial factor influencing the willingness of FSWs to register. Internationally, due to the quasi-criminalisation and stigmatisation of sex work, FSWs are often forced to hide their involvement in sex work from family, friends, and their home communities [20]. It is known that breaches of confidentiality can lead to adverse consequences, including stigmatisation and discrimination of FSWs [21]. Despite the implementation of safeguards to uphold a registered FSW confidentiality, some potential breaches were identified. Firstly, some hospitals designate specific days for FSWs to attend their compulsory health visits, making them discernible to the general hospital population. Additionally, it was reported that there was differential treatment by health facility staff, a challenge not unique to Senegal, which could potentially expose FSWs’ activities publicly [22]. Addressing these concerns entail integrating FSWs’ health visits with general consultations or women’s/maternity health services and promoting ongoing education on the significance and methods of ensuring confidentiality. The DCE revealed that confidentiality ensured at the health centre level emerged as the most salient policy attribute for both groups of FSWs [15]. Registered FSWs demonstrated a 26 percentage point preference for policies guaranteeing anonymity at the health centre, compared to those that do not. Similarly, non-registered FSWs exhibited a 22.1 percentage point preference for confidentiality.

The second issue revolves around the registration booklet FSWs must carry to validate their legal work status. This card serves as an identification that effectively labels them as FSWs, raising fears among FSWs of potential exposure if the card is discovered by others. As founded in previous studies, the associated stress, coupled with the risk of severe social stigmatisation, compels FSWs to conceal these cards from friends and family [7]. Replacing the physical card with a less accessible form of identification, such as a QR code, was proposed as a solution. A QR code would not hold identifiable information and therefore would hold minimal significance for the public. According to the findings of the DCE, the adoption of a QR code for proof of registration, in contrast to the current ’carnet sanitaire,’ would enhance the likelihood of preferring a policy by 5.9 percentage points among registered FSWS and by 3.5 percentage points among non-registered FSWs [15].

The implementation of a QR code as FSW identification would mark the inaugural trial of its kind internationally. The feasibility of deploying such an intervention was discussed with government officials, police representatives, and leaders within the sex worker community. Overall, there was a consensus that this initiative would align with Senegal’s ongoing computerisation of patient health records. Furthermore, the integration of QR code training for healthcare personnel could seamlessly become part of broader digital health reforms. The introduction of portable scanners for policemen to scan FSWs’ QR codes received positive feedback from both police representatives and leaders among FSWs. Police stakeholders suggested that the use of QR codes could act as a deterrent to bribery, a practice previously reported where police officers created and sold fraudulent registration cards to FSWs. The use of QR codes would make such illegal activities much more difficult. The main challenge identified by policy informants was the allocation of resources to purchase the necessary materials, which was a significant barrier to implementing this policy change.

As mentioned, the storage of health files at the facility level caused distress for FSWs who felt that their confidential information was inadequately protected. These files, typically stored in unlocked cabinets, raised concerns about unauthorised access. Furthermore, FSWs observed that even after leaving sex work, their health files were retained at the health facility level. Health staff justified this practice by noting that FSWs often take short breaks rather than completely discontinuing their sex work careers. Retaining health files allows for the resumption of medical visits and health services without loss of relevant information upon their return to sex work. However, even in cases of actual interruptions, FSW health files are archived and persist at the health centre level. The police similarly lacked confirmation on whether FSWs’ files were destroyed at the police station upon leaving sex work. This storage practice poses a potential risk to confidentiality. These findings likely explain why registered FSWs demonstrated a 14.6 percentage point increase, while non-registered FSWs showed a 17.0 percentage point increase in likelihood to prefer a policy where their health file is exclusively retained at the health centre level, compared to the current practice of their files being held both at a health centre and by the police indefinitely, as determined by the DCE results [15]. Transitioning to electronic medical records was identified as a mitigation strategy to address future risks of confidentiality breaches.

Furthermore, another recurrent theme that arose was that both registered and non-registered FSWs possess inadequate and inaccurate information regarding the registration policy and its potential benefits. This occurs from a combination of circulating rumours and the uncertainty within the institutions themselves regarding the applicable rules and regulations, leading to the prevailing situation wherein a majority of FSWs opt to not register. For registered FSWs, inadequate information provided during the registration process by hospital staff contributes to the perpetuation of these misconceptions. For example, a lack of awareness among certain policy actors about the registration file storage policy appears to be a significant factor contributing to the absence of information provision at the time of registration. The ambiguous responses obtained during interviews add to the challenge of comprehending the extent of communication or file exchange between hospital and police staff. This uncertainty mirrors the broader issue of inadequate communication between these institutions, underscoring the evident reason why FSWs often find themselves devoid of clear and accurate information.

An additional significant concern identified pertains to the dynamic between FSWs and the police. Criminalisation of sex work creates a substantial power imbalance, globally recognized for fostering police abuse against FSWs, encompassing improper arrests, detentions, and instances of economic, sexual, and physical abuse [23, 24]. In the specific case of Senegal, the possession of a registration booklet appears to amplify the vulnerability of FSWs to police abuse. Mirroring patterns observed within the global sex work community, younger FSWs in Senegal are increasingly transitioning to online solicitation through dating apps and social networks, steering away from street-based work. This strategic shift globally aims to reduce the likelihood of detection, harassment, and to enhance overall safety of both male and FSWs [25, 26]. Consequently, this change in solicitation practices diminishes the perceived advantages of registration for many, as they distance themselves from traditional street solicitation, where the possession of a registration booklet is a prerequisite for legal solicitation.

Finally, it is evident, supported by responses from policy stakeholders, that a significant shortcoming in the current registration policy is the absence of psychological support to address the prevalent mental health issues among FSWs. A recent systematic review focusing on the on mental health in low- and middle-income countries revealed concerning pooled prevalence rates: 41.8% for depression, 21.0% for anxiety, 19.7% for Post-Traumatic Stress Disorder (PTSD), and 40.8% for psychological distress [27]. The high rates of mental health problems are a consequence of physical and psychological violence from clients, peers, and law enforcement, contributing to a pervasive environment of vulnerability [28]. Moreover, our findings shed light on the immense stress endured by FSWs due to the imperative of concealing their activities from friends and family. This intricate web of challenges highlights the multifaceted nature of the mental health struggles faced by FSWs, a phenomenon well-documented beyond Senegal’s borders [13, 21]. The DCE founded that psychological support was a crucial element for improving the rate of registration of FSWs, with its provision boosting the likelihood of policy preference among registered and non-registered FSWs by 5.1 and 5.7 percentage points, respectively [15]. Our interviews also revealed that health staff and some sex work leaders are cognizant of these challenges and have attempted to address the gap by assuming the role of psychological counsellors, despite constraints in time, training, and resources. However, in alignment with the global commitment to universal health coverage, as outlined in Goal-3 of the Sustainable Development Goals, our respondents put forth a compelling argument for the integration of a state-funded psychological support service that is universally accessible to all FSWs [29].

There are several limitations to this study. Our main constraint was the number of participants interviewed to represent each group. Despite our best efforts to include a diverse range of representatives to ensure a broad spectrum of opinions, the reality is that only a few individuals could represent each group. Consequently, it is challenging to assume that the views expressed by the participants are reflective of the entire group or organisation they represent. Furthermore, our ability to conduct interviews was constrained by the limited time available to key informants, who often held busy positions such as doctors or worked in bustling hospitals. Additionally, certain interviewees felt hesitant to express themselves openly due to the sensitivity of the topics discussed; speaking in a manner deemed inappropriate could potentially jeopardise their reputation and professional advancement. While we ensured complete confidentiality for our interviewees to minimise any discomfort, there may still have been some reluctance in their responses.

## 5. Conclusion

This study provides a comprehensive understanding of the challenges and opportunities within Senegal’s unique context of sex work regulation, with implications for sex work legislation globally. This study examines the possibilities presented by policy adjustments within the registration policy tailored specifically for FSWs, as identified in previous research. The findings underscore the need for nuanced and comprehensive policy reforms that address the multifaceted issues faced by FSWs. While FSWs registration as a public health policy has the potential to limit STIs and consequently HIV acquisition and transmission, the current issues in the design and implementation of the policy in Senegal explains the current low proportion of registered FSWs. The study showed that several policy changes have the potential to increase the registration rate of FSWs and improve their wellbeing without overturning the law and were supported by key national stakeholders. For example, changing the registration proof to a QR code, bettering relationships and reinforcing dialog between FSWs and police officers, improving the dissemination of correct information regarding the benefits and costs of registration and including psychosocial support in the registration package could generate important incentives for FSWs to register. In addition, integrating FSWs’ health visits with maternal health appointments and reinforcing training of health workers to ensure confidentiality in health centres have the potential to reduce FSWs concerns about breaches of confidentiality, a major risk associated with registration.

## Supporting information

S1 Appendix Number of interviewees for qualitative study. (DOCX)

S1 File Financial disclosure. (DOCX)

S2 File Inclusivity in global research. (DOCX)
