# Supplementary material for: Optimising the public health benefits of sex work regulation in Senegal: Results from qualitative interviews with policy stakeholders
Source: PLoS One. 2024 Aug 15;19(8):e0306803. doi: 10.1371/journal.pone.0306803 (PMC11326597; doi:10.1371/journal.pone.0306803)
Supplement: S1 Appendix — (DOCX) [file pone.0306803.s001.docx]

**Appendix 1: Number of interviewees for qualitative study**

| **Respondent type** | **Number** |
| --- | --- |
| Nurse / Midwife | 4 |
| Group leader in sex work | 3 |
| NGO director – key population focus | 3 |
| District head doctor | 4 |
| Members of police | 2 |
| Government representatives | 4 |
| Key population government representative | 2 |
| **Total** | **22** |
