# Supplementary material for: Optimising the public health benefits of sex work regulation in Senegal: Results from qualitative interviews with policy stakeholders
Source: PLoS One. 2024 Aug 15;19(8):e0306803. doi: 10.1371/journal.pone.0306803 (PMC11326597; doi:10.1371/journal.pone.0306803)
Supplement: S1 File — (DOCX) [file pone.0306803.s002.docx]

**Financial disclosure**

This research was funded by the UK Medical Research Council. The funders had no role in study design, data collection and analysis, decision to publish, or preparation of the manuscript
